# An inhibitory role of progerin in the gene induction network of adipocyte differentiation from iPS cells

**DOI:** 10.18632/aging.100550

**Published:** 2013-04-14

**Authors:** Zheng-Mei Xiong, Christina LaDana, Di Wu, Kan Cao

**Affiliations:** Department of Cell Biology and Molecular Genetics, University of Maryland, College Park, MD 20742, USA

**Keywords:** adipogenesis, aging, lamin A, Progeria, iPSC

## Abstract

Lipodystrophies, characterized by partial or complete loss of adipose tissue, have been associated with mutations in the lamin A gene. It remains unclear how lamin A mutants interfere with adipose tissue formation. Hutchinson–Gilford progeria syndrome (HGPS) presents the most severe form of lamin A-associated diseases, whose patients show a complete loss of subcutaneous fat. Using iPSCs reprogrammed from HGPS fibroblasts, we induced adipocyte formation from iPSC derived embryoid bodies or from iPSC derived mesenchymal stem cells. Both approaches revealed a severe lipid storage defect in HGPS cells at late differentiation stage, faithfully recapitulating HGPS patient phenotype. Expression analysis further indicated that progerin inhibited the transcription activation of PPARγ2 and C/EBPα, but had little effects on the early adipogenic regulators. Our experiments demonstrate two comparable approaches of *in vitro* modeling lipodystrophies with patient-specific iPSCs, and support a regulatory role of lamin A in the terminal differentiation stage of adipogenesis.

## INTRODUCTION

Adipocytes are essential regulators of whole-body energy homeostasis. Compared to other cell types with esterified lipids, these cells are unique in the large quantity of lipids that they can store. In addition, adipocytes secrete proteins that regulate diverse processes including blood pressure control, immune response, angiogenesis and reproductive function [1-3]. Partial or complete loss of adipose tissue characterizes a growing group of genetic disorders known as lipodystrophies [1, 4, 5].

Two major steps are involved in adipocyte formation, including initial determination and terminal differentiations. The determination phase involves the commitment of a pluripotent or multipotent cell to adipocyte lineage. During the terminal differentiation phase, the pre-adipocyte develops the characteristics of a mature adipocyte, including the accumulation of large lipid droplets and the conversion to a rounded cell shape (Rosen and MacDougald, 2006). At a molecular level, adipocyte differentiation is controlled by a temporal transcription induction of a set of adipogenic genes, including the early differentiation regulators C/EBPβ and C/EBPδ and two master regulators PPARγ and C/EBPα for establishing and maintaining the terminal adipocyte differentiation stage [2, 6]. The induction of C/EBPβ and C/EBPδ at the early differentiation stage promotes the transcription activation of the central regulators C/EBPα and PPARγ, and this action is partially accomplished by binding of C/EBPβ and C/EBPδ to their promoters [2, 6]. Moreover, lipodystrophies have been associated with mutations in *LMNA*, the gene that encodes A-type lamins. Mutations in *LMNA* have been associated with various diseases with lipodystrophic phenotypes, including Dunnigan type familial partial lipodystrophy, mandibuloacral dysplasia and atypical Werner's syndrome [5, 7-9]. Inversely, suppression of lamin A in mouse models and in cultured cells promotes adipocyte lineage commitment [10, 11]. The relationships between A-type lamins and various C/EBP proteins and PPARγ remain unclear [4].

Lamins belong to type V intermediate filament proteins and are the main components of the nuclear lamina [5, 12, 13]. Based on sequence homologies in mammals, there are two major A-type lamins (lamins A and C) encoded by the *LMNA* gene with alternative splicing, and two major B-type lamins (lamin B1 and B2) encoded by *LMNB1* and *LMNB2*, respectively. Lamin B proteins are expressed throughout development and are essential for cell survival, whereas lamins A and C usually appear only in specific tissues and organs [14-19].

At least 16 distinct diseases have been linked to mutations in *LMNA* gene, collectively known as laminopathies [5]. One of the laminopathies that exhibits a very severe lipodystrophic symptom is Hutchinson-Gilford progeria syndrome (HGPS), whose patients show a complete loss of subcutaneous fat [20]. HGPS is a rare dominant genetic disease caused by a single-base substitution, C1284T, in the exon 11 of *LMNA* [21]. This mutation results in the activation of a cryptic splice donor site that yields a mutant protein with a 50 amino acid deletion near the carboxyl terminus. This mutant is termed progerin [21]. The presence of progerin in the nuclear lamina leads to abnormal nuclear morphology (or nuclear blebbing), which has been noted as the cellular hallmark of HGPS cells [21-26].

To study the function of lamin A in adipocyte differentiation, we generated induced pluripotent stem cells (iPSCs) from normal and HGPS primary skin fibroblasts and examined adipocyte differentiation directly from iPSC derived embryoid bodies (EBs) or from iPSC derived mesenchymal stem cells (MSCs). We found that these two distinct methods revealed consistent results. The expressions of lamin A/C and progerin were absent in iPSCs and up-regulated in the presence of adipogenic stimuli. Correlatively, we observed a significant reduction in lipid storage in HGPS adipocytes compared to normal adipocytes, as well as characteristic HGPS cellular phenotypes including nuclear blebbing, binucleation, and premature senescence. Live cell lipid analysis suggested that the HGPS cells appeared to respond to the adipogenic stimuli during early differentiation, but they failed to commit to the late adipogenic stage. In support, expression array analysis indicated that progerin specifically repressed a subgroup of adipogenic regulators, including the two core players PPARγ2 and C/EBPα, but has little inhibitory effect on the activation of the early adipogenic regulators C/EBPβ and C/EBPδ. Our experiments support an inhibitory role of progerin in controlling late stage gene induction network during adipogenesis.

## RESULTS

### Absence of A-type lamins in iPSCs

**Figure 1:**
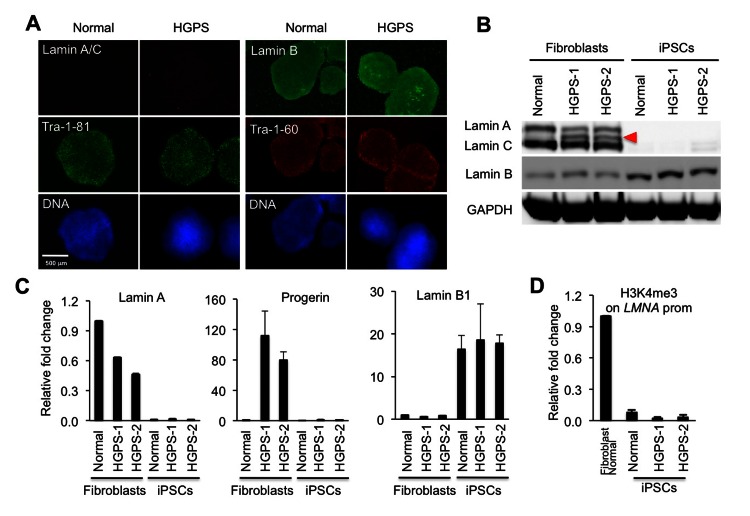
Absence of A-type lamins in iPSCs is associated with low levels of H3K4me3 on the *LMNA* promoter (**A**) Immunofluorescence staining in normal and HGPS iPSCs with stem cell markers Tra-1-81 (green) and Tra-1-60 (red), and with lamin A/C antibody (red) and lamin B antibody (green). Nuclei are counterstained in blue with Hoechst 33342. Scale bar: 500 μm. (**B**) Western Blotting analysis with lamin A/C, lamin B, and GAPDH antibodies in normal and HGPS fibroblasts and iPSCs. Red filled triangle points to progerin. (**C**) Quantitative RT?PCR analysis showing the mRNA level of lamin A (left), progerin (middle), and lamin B (right) in normal and HGPS fibroblasts and iPSCs (n = 3). (**D**) ChIP-qPCR analysis of H3K4me3 at the LMNA gene promoter in a normal fibroblast line and three iPSC lines (n = 3). Cell lines: Normal (8470), HGPS or HGPS1 (164), and HGPS-2 (155).

It has been shown that embryonic stem cells (ESCs) can be differentiated into adipocytes with a combination of retinoic acid and pro-adipogenic hormones [6]. To set up an *in vitro* cellular model of HGPS, we generated iPSCs from two HGPS primary skin fibroblast lines (HGADFN164: HGPS-1 and HGADFN155: HGPS-2, respectively) and one age-matched normal fibroblast line (AG08470) by retroviral transduction of *KLF4, SOX2*, *OCT4*, and *C-MYC* cocktails [27, 28] (See table S1 for cell line information). Characterization of all three iPSC lines showed an up-regulation of telomerase protein subunit (Tert) and various pluripotent markers, including Nanog, Oct4, SSEA4, Tra-1-60 and Tra-1-81 (Figures S1A and S1B). Alkaline phosphatase (AP) staining further confirmed the undifferentiated state of these iPSC colonies (Figure S1B). Consistent with previous reports [15, 16, 29], we found that the expression of lamin A/C and progerin was absent in both control and the two HGPS iPS cell lines (Figures 1A, B and C). In accordance, Chromatin Immuno-precipitation-coupled with quantitative PCR (ChIP-qPCR) with primers for *LMNA* gene promoter showed that H3K4me3, an epigenetic marker on the promoter of actively transcribed genes, was absent in the iPSCs (Figure 1D). Interestingly, lamin B, the other major component in the nuclear lamina, was up-regulated in the iPSCs compared to parent fibroblasts (Figures 1B and 1C). Chromosome karyotype analysis revealed that both the normal control and HGPS-1 iPSC lines carry normal karyotypes (Figures S1C and S1D), but HGPS-2 cells showed signs of possible chromosome translocation between chromosomes 2 and 22 (Figures S1E and S1F). Thus, we only used normal (8470) and HGPS-1(164) for adipocyte differentiation study.

### Adipocytes differentiated from HGPS iPSC derived EBs show defective lipid storage

**Figure 2:**
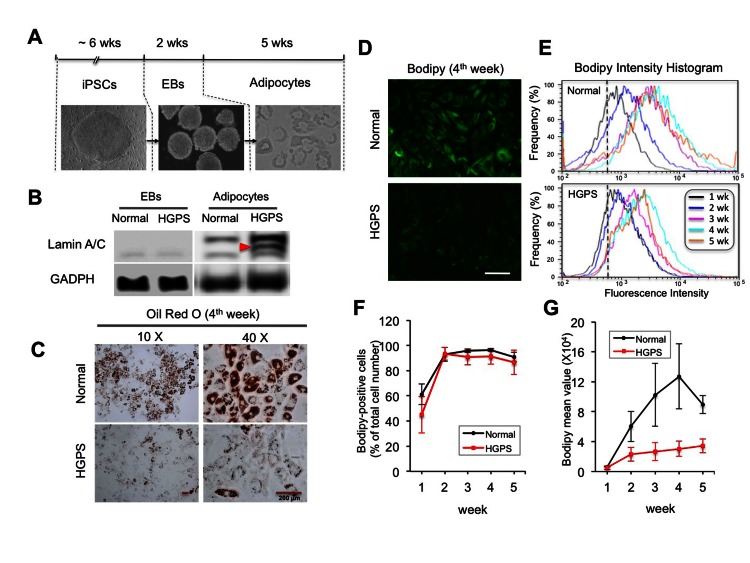
Adipocytes differentiated from HGPS iPSC derived EBs show defective lipid storage (**A**) Schematic diagram of the experimental timeline starting from iPSCs, via intermediate stage EBs, into the differentiated adipocytes with phase contrast pictures underneath each stage. (**B**) Western Blotting analysis with lamin A/C and GAPDH antibodies in normal and HGPS EBs and adipocytes. Red filled triangle points to progerin. (**C**) Images of Oil red O staining show red lipid droplets in normal and HGPS adipocytes at the fourth week of adipogenic differentiation. (**D**) Images of bodipy 493/503 fluorescence staining in normal and HGPS adipocytes at the fourth week of adipogenic differentiation. (**E**) Representative histograms of fluorescence intensity of bodipy 493/503 staining during five-week adipocyte differentiation. (**F**) The percentage of bodipy positive cells from three independent experiments. (**G**) The mean fluorescence intensity of bodipy staining in normal and HGPS adipocytes during the five weeks of adipogenesis from three independent experiments. Cell lines: Normal (8470) and HGPS (164).

To model lipodystrophy in HGPS, we first explored the adipocyte differentiation from iPSC derived EBs (Figure 2A). At the EB stage, we observed a slight but significant increase in lamin A/C expression compared to iPSCs (Figure 2B and Figure S2A). In adipocytes, the lamin A/C expression was restored to a level comparable to that in fibroblasts (Figure 2B and Figure S2A). The expression of progerin was also significantly increased, but only in HGPS adipocytes as expected (Figure 2B and Figure S2A). Phenotypically, in the first week of induction, comparable amounts of lipid droplets in both normal and HGPS cells were observed (Figure S2B). However, starting from the second week, fewer oil droplets were seen in HGPS cells compared to the normal cells (Figure S2B). To quantify lipid storage, Oil Red O (ORO) staining was measured in normal and HGPS adipocytes, which revealed at least 50% reduction in ORO value in HGPS compared to normal at the fourth week (Figure 2C, *p*<0.01 from three independent repeats).

To monitor the lipid amount during induction, a fluorescent lipid dye Bodipy 493/503 was used to stain live cells during the five weeks of induction, and the fluorescence signals were quantified by an automated flow cytometry (Figures 2D and 2E). Analysis revealed that the majority of cells in both normal and HGPS samples showed positive Bodipy signals starting from the second week of induction (Figure 2F), suggesting that these cells have committed to the initial adipogenic stage. The intensity of Bodipy signal reflects the amount of lipids in each sample. When the mean intensities were plotted against time, we found that the amount of lipids progressively increased with time in normal adipocytes and peaked at the fourth week (Figure 2G). However, in HGPS adipocytes, the increase was insignificant (Figure 2G). Together, these results suggest that while the HGPS cells appeared to respond to the adipogenic stimuli during differentiation, they failed to commit to the terminal differentiation stage.

### HGPS adipocytes exhibit characteristic cellular phenotypes

**Figure 3:**
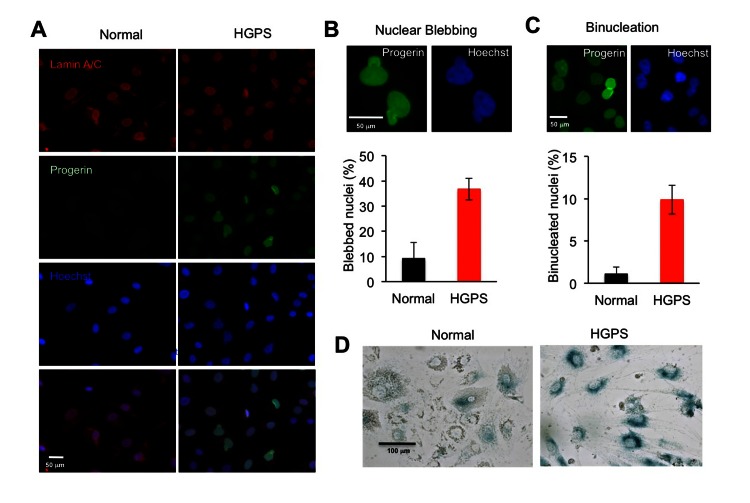
HGPS adipocytes exhibit characteristic cellular phenotypes (**A**) Immunofluorescence images of normal and HGPS adipocytes with lamin A/C staining in red and progerin staining in green at the fourth week differentiation. Nuclei are counterstained in blue with Hoechst 33342. Scale bar: 50 μm (**B**) Upper panel: Representative images of blebbed nucleus stained with progerin antibody and Hoechst 33342. Lower panel: Histogram shows the percentage of blebbed nuclei. (**C**) Upper panel: Representative images of binucleated nuclei stained with progerin antibody and Hoechst 33342. Lower panel: Histogram shows the percentage of binucleated nuclei. (**D**) Senescence-associated (SA)-β-Gal stainings of normal and HGPS adipocytes at the fourth week differentiation shows premature cellular senescence. Scale bar: 100 μm. Cell lines: Normal (8470) and HGPS (164).

To characterize the cellular phenotypes during adipocyte differentiation, we performed immunostaining with anti-lamin A/C and anti-progerin antibodies [23]. 100% of cells in both normal and HGPS samples were positively stained with the lamin A/C antibody (Figure 3A). In addition, all HGPS adipocyte showed positive signals for progerin (Figure 3A). Similar to the primary HGPS fibroblasts [24], the expression of progerin in these cells led to increased nuclear blebbing, the hallmark of HGPS cellular phenotype. Over 30% of HGPS adipocytes showed nuclear blebbing compared to about 10% in normal adipocytes at the fourth week of differentiation, according to the blinded counting of over 200 cells by independent observers (Figure 3B). Consistent with previous reports of mitotic abnormalities in HGPS fibroblast cells [30, 31], we also found a five-fold increase in binucleated cells in HGPS adipocytes (Figure 3C), which suggests abnormal chromosome segregation during adipocyte differentiation at the presence of progerin. Moreover, progerin-expressing adipocytes exhibited premature cellular senescence as demonstrated by the increased senescence-associated- β -galactosidase (SA–β-gal) staining (Figure 3D). Collectively, we found that the progerin-expressing adipocytes differentiated from HGPS iPSCs exhibited characteristic HGPS cellular defects as previously reported in HGPS fibroblasts [30].

### Progerin suppresses the activation of core adipogenic regulators

**Figure 4:**
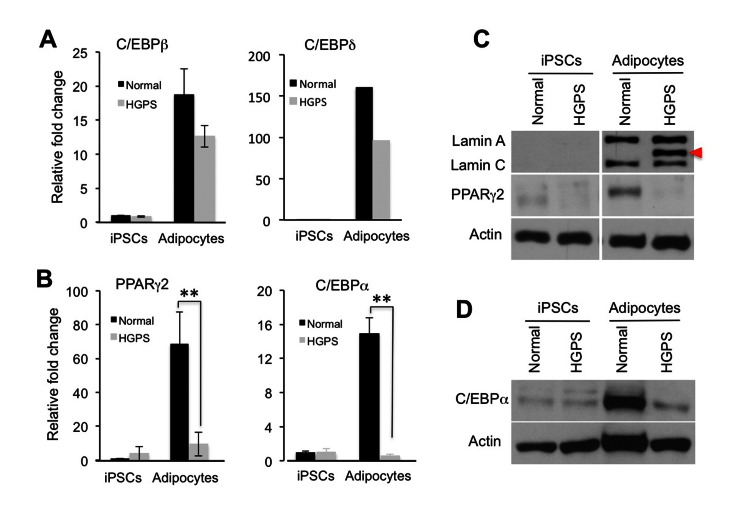
Progerin suppresses the transcription activation of core adipogenic regulators during adipocyte differentiation (**A**) and (**B**) Quantitative RT-PCR analysis of early adipogenic genes C/EBPβ and C/EBPδ (**A**) and late adipogenic genes PPARγ2 and C/EBPα (**B**) in normal and HGPS iPSCs and adipocytes. n = 3. ** *p* < 0.01. (**C**) Western Blotting analysis with lamin A/C, PPARγ2, and actin antibodies in normal and HGPS iPSCs and adipocytes. Red filled triangle points to progerin. (**D**) Western Blotting analysis with C/EBPα and actin antibodies in normal and HGPS iPSCs and adipocytes. Cell lines: Normal (8470) and HGPS (164).

To understand how progerin interferes with adipocyte differentiation, we collected RNA samples from the normal and HGPS adipocytes and performed gene expression analysis on the core adipogenic regulators PPARγ2, C/EBPα, C/EBPβ and C/EBPδ using quantitative RT-PCR. In agreement with the lack of phenotypes in the first week of adipocyte differentiation (Figure S2B), we found that the expression of C/EBPβ and C/EBPδ, two early adipogenic transcription factors, were up-regulated in both normal and HGPS samples (Figure 4A). In contrast, PPARγ2 and C/EBPα, the two master regulators for the terminal adipocyte differentiation, were only activated in normal adipocytes (Figure 4B). Western blotting analysis further verified the down-regulation of PPARγ2 and C/EBPα proteins in HGPS samples (Figures 4C and 4D).

### Adipocytes differentiated from HGPS iPSC derived MSCs show similar defects

**Figure 5:**
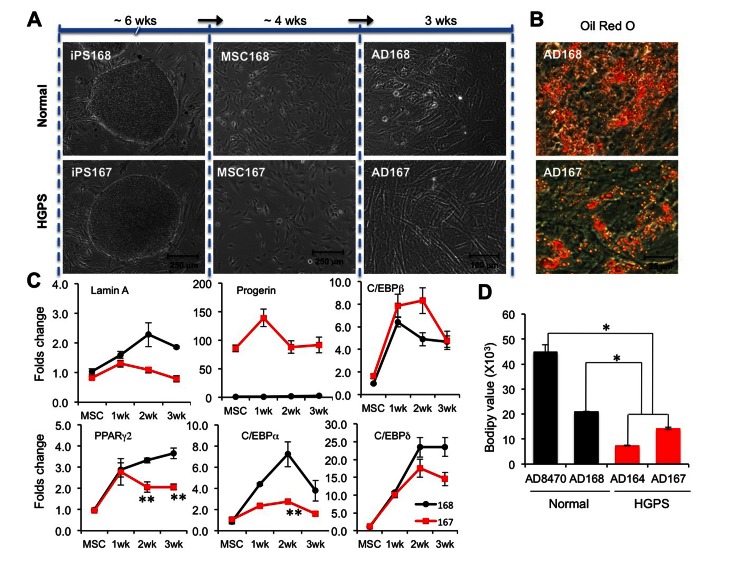
Adipocytes differentiated from HGPS iPSC derived MSCs show similar defects (**A**) Schematic diagram of the experimental timeline starting from iPSCs, via MSCs, into ADs (adipocytes) with phase contrast pictures underneath each stage. Cell lines: Normal (168) and HGPS (167). (**B**) Images of Oil red O staining show red lipid droplets in AD168 and AD167 adipocytes at the third week of adipogenic differentiation. (**C**) Quantitative RT-PCR analysis of lamin A, progerin, early adipogenic genes C/EBPβ and C/EBPδ, and late adipogenic genes PPARγ2 and C/EBPα in normal AD168 (black) and HGPS AD167 (red) cells during three weeks adipogenesis from MSCs. n = 3. *p < 0.05, ** *p* < 0.01. (**D**) Fluorescence intensity of bodipy 493/503 staining at the third week of adipocyte differentiation. *p < 0.05.

Previous studies using mesenchymal stem cells (MSCs) to study adipogenic lineages in HGPS have reported inconsistent results [16, 32]: Scaffidi and Misteli showed a significant reduction in ORO signals in adipocytes differentiated from the hMSCs ectopically expressing GFP-progerin; in contradiction, Zhang and colleagues did not find any impacts of progerin on adipogenesis using patient-specific MSCs. To address this discrepancy, we generated MSCs from two additional iPS cell lines (Normal-168 and HGPS-167) with a one-step differentiation protocol (see Experimental Procedures for details). These iPSC derived normal and HGPS MSCs were then labeled and sorted with positive (CD90) and negative (CD45) markers for MSC (Figure S3A). Consistent with a previous study [16], expression analysis revealed high levels of lamin A and progerin expression in these MSCs (Figure 5C and Figure S3B). Next, adipocytes were induced from iPSC derived MSCs by treatment of a combination of adipogenic stimuli (See Experimental Procedures). After three weeks of induction, more than 50% of normal MSC168 adapted a rounded morphology (Figure 5A). However, most of the HGPS MSC167 (over 95%) displayed an elongated spindle-like shape, indicating that they failed to commit to the terminal adipogenic stage (Figure 5A). Consistently, ORO staining revealed significantly fewer lipids in HGPS AD167 than in normal AD168 cells (Figure 5B). To assay the effects of progerin on gene expression, the expression of four core adipogenic regulators was monitored weekly during the three weeks of induction. Again, we found that the expression of the early adipogenic genes C/EBPβ and C/EBPδ was up-regulated in HGPS samples at the levels comparable to the controls, and the transcription activation of C/EBPα and PPARγ2 was suppressed in HGPS (Figure 5C). Together, these data suggest that the transcription suppression of PPARγ2 and C/EBPα by progerin is a common mechanism in both EB- and MSC-mediated adipocyte differentiations from HGPS iPSCs.

Of the four iPSC lines tested in both methods (Normal 8470, Normal 168, HGSP164, and HGPS167), only the donor of normal168 was an adult and the other donors were under 10 years old (Table S1). It is unclear whether the adipocyte differentiation potential can be influenced by donor age. In an attempt to address this, we compared the lipid amounts in all four lines (Normal-8470, Normal-168, HGPS-164, and HGPS-167) during a three-week induction with iPSC-EB method (Figure 5D). Interestingly, while both normal cells showed cell shape changes, the normal 168 cells had fewer lipids than normal 8470 whose donor was a 10-year old (Figure 5D). This observation suggests a potential influence from the age of the donors in adipogenesis. Importantly, despite the difference in normal donor ages, the HGPS adipocytes showed significantly fewer lipids compared with either controls (Figure 5D, *p*<0.05).

### Progerin affects a subgroup of genes in adipogenic network

**Figure 6:**
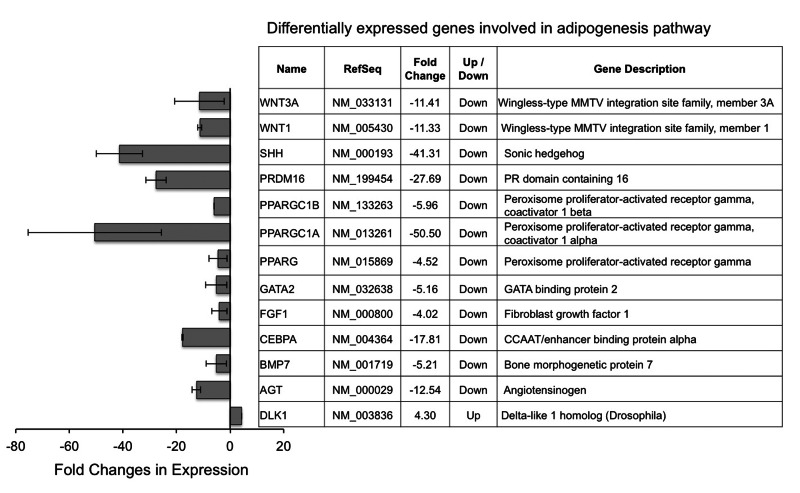
Differentially expressed adipogenic genes in HGPS adipocytes compared to normal adipocytes Cell lines: Normal (8470) and HGPS (164).

Finally, we asked whether progerin induces a broader set of gene expression changes in adipogenic network, beyond its effects on PPARγ and C/EBPα. To answer this question, we employed the human adipogenesis PCR array, which probes 84 genes known to be involved in the differentiation and maintenance of mature adipocytes. The complete gene list is available through the link: http://www.sabiosciences.com-rt_pcr_product/HTML/PAHS-049Z.html. The comparative analysis revealed a list of 12 out of the 84 genes showed over four folds down-regulation in HGPS adipocytes compared to normal cells, which includes not only PPARγ2 and C/EBPα, but also two PPARγ coactivators (PGC1α and PGC1β) and a downstream effector of PPARγ (AGT, angiotensinogen, Figure 6) [33]. Several additional proadipogenic genes are significantly inhibited, including fibroblast growth factor 1 (FGF1), bone morphogenetic protein 7 (BMP7), and PR domain containing 16 (PRDM16)[34-36]. Moreover, DLK1, an inhibitor for adipogenesis, showed a four-fold up-regulation in HGPS samples (Figure 6)[37]. Surprisingly, we also found four negative regulators of adipogenesis, including sonic hedgehog (SHH), GATA binding protein 2 (GATA2), WNT1, and WNT3A [38-40]. The undermined anti-adipogenesis signaling might be explained by a possible negative feedback system that is triggered by severe lipid storage defects in HGPS adipocytes.

## DISCUSSION

Recently, several groups showed the absence of lamin A/C expression in iPSCs [15, 16, 29]. In accordance with their reports, we found the silencing of the *LMNA* gene transcription in iPSCs, which was coupled with a reduction in H3K4me3 on the promoter of *LMNA* (Figure 1). Methylation study indicated *LMNA* gene silencing in iPSCs was not controlled by DNA methylation [15]. Interestingly, a recent study showed a brain specific microRNA (MiR-9) to be responsible for the down-regulation of lamin A but not lamin C in neurons [17]. Future work is required to decipher the regulation of lamin A expression in ESCs /iPSCs. In this study, we showed that lamin B was up-regulated in iPSCs in the absence of lamin A/C, implying a certain total amount of lamin proteins may be needed in iPSCs. It is intriguing since mouse knockout analysis suggests that lamins are not needed for self-renewal and pluripotency in mouse ESCs [14]. The questions of how and why lamin B is up-regulated in iPSCs remain to be explored.

Due to the rarity of HGPS cases, pathological analyses have been quite limited [41, 42]. Much of information relating to the pathologies of HGPS has come from the studies using patient skin fibroblasts [24, 30] and cell lines that ectopically express progerin [24, 30]. Some progeria mouse models successfully recapitulated the patient adipose phenotype [9, 43, 44], however it remains unknown how lamin A mutants interfere with adipocyte differentiation process. The patient-specific iPS technologies provide new opportunities to *in vitro* model the HGPS disease pathologies as well as other rare laminopathies [45, 46]. In this study, we examine the adipocyte differentiation with two approaches: the iPSC derived EBs and the iPSC derived MSCs. Both studies reveal a lipid storage defect that faithfully recapitulates the lipodystrophy phenotype in HGPS patients (Figures 2 and 5). In addition, our data reveal characteristic cellular abnormalities in differentiated HGPS cells including nuclear blebbing and binucleation (Figure 3). Adipogenesis is controlled by a cascade of fat cell-related transcriptional factors [6]. Using the powerful iPSC *in vitro* system, we find that the presence of progerin suppresses the induction of PPARγ2 and C/EBPα during adipogenic induction, but does not appear to affect the expression of two early adipogenic factors C/EBPβ and C/EBPδ (Figures 4 and 5). Significantly, these gene expression changes in HGPS samples precisely match the phenotypic development, which, at first, appears normal but becomes progressively defective at the terminal differentiation stage (Figures 2, 4 and 5). To our knowledge, this is the first HGPS study that links alternations in gene induction network to the phenotype manifestation during *in vitro* adipocyte differentiation. Our study suggests a regulatory role of lamin A in gene induction network at the terminal adipocyte differentiation stage.

In addition, high-throughput adipogenesis array analysis reveals that progerin specifically interferes with a subgroup of late adipogenic regulators (Figure 6). We do not yet have a molecular explanation to address how progerin interferes with this subgroup of adipogenic genes. One possibility is that progerin interacts with an intermediate transcription regulator(s) that regulates a set of genes with certain motifs. In support to this, a recent studies suggested FOXQ1, a transcriptional factor, can function as a potential effector of progerin [47]. The other possibility is that progerin may directly inhibit an essential adipogenic gene via physical interaction, whose mis-regulation triggers downstream alterations in transcription. Future analysis will be required to answer this question. Moreover, our data provides evidence that the age of donors may play a role in influencing the adipocyte differentiation potentials from patient-specific iPSCs (Figure 5D), which is consistent with a recent report on a decline in responsiveness in adipogenesis as a function of age [48]. Given the increasing involvement of lamin A/progerin in the normal aging process [42, 49-54], experiments with a combination of iPSC technologies and high-throughput analysis may reveal new connections between adipogenic potential decline and aging.

## METHODS

### Cell Culture

For reprogramming to iPSCs, the normal human skin fibroblast line AG08470 (normal) was obtained from Coriell, and two HGPS fibroblasts HGADFN164 (HGPS-1) and HGADFN155 (HGPS-2) were from the Progeria Research Foundation (PRF). Both HGADFN164 and HGADFN155 carry the classic C1824T mutation. The two additional iPS lines 168 (Normal) and 167 (HGPS) were obtained from PRF (also see Table S1).

All fibroblast cell lines were cultured in MEM (Life Sciences) supplemented with 15% FBS (Gemini Bio-Products) and 2 mM L-glutamine (Life Sciences) at 37°C with 5% CO_2_. Induced pluripotent stem cells (iPSCs) were cultured on irradiated mouse embryonic fibroblasts (iMEFs, R&D) feeder cells in DMEM/F12 (Lonza) supplemented with 20% knockout serum replacement (KSR, Life Sciences), 1X nonessential amino acid (Life Sciences), 100 mM 2-mercaptoethanol (Life Sciences), 1 mM L-glutamine (Life Sciences), and 5 ng/ml rhFGF (R&D). Embryoid bodies were cultured in the same iPSCs medium without rhFGF. Adipocytes were induced and maintained in DMEM/F12 (Lonza) plus10% KSR supplemented with 1 mg /ml insulin (TOCRIS), 0.5 mM 3-isobutyl-1-methylxan-thine (IBMX), 0.25 mM dexamethasone, 0.2 mM indomethacin, and 1 mM pioglitazone (all from Sigma).

### Retroviral Production and iPSCs Generation

The pMIG retroviral vectors encoding the human cDNAs for *KLF4, SOX2*, *OCT4*, and *C-MYC* were generously provided by Dr. George Daley (Harvard). For retrovirus production and iPSCs generation, the protocol described by Park et al. was followed [27]. Briefly, pMIGs plasmids were cotransfected with packaging plasmid gag-pol and VSV-g into BOSC23 packaging cells using Fugene 6 (Promega). Viral supernatant fractions were collected after 72-hour incubation, filtered through a 0.45 μm filter, and concentrated by ultracentrifugation. 50,000 fibroblast cells were infected with OCT4, SOX2, KLF4, and MYC retroviruses at a multiplicity of infection (MOI) of 5 using 5μg/ml polybrene (Santa Cruz). After four days, infected cells were transferred onto iMEFs and cultured in iPSCs medium. The medium was changed every other day. The iPS colonies with clear boundary were manually picked after two to three weeks.

### Differentiation of iPSCs into Adipocytes via EB formation

We follow the previously described adipocyte differentiation protocol [55] with minor modifications. Briefly, the iPS colonies were cut into small pieces using StemPro EZPassage (Life Sciences) and plated onto non-adherent culture dishes (Falcon), where they were floating in maintenance medium without rhFGF. After two weeks, EBs were gently trypsinized in 0.05% trypsin-EDTA (Life Sciences) for 5 minutes. After neutralization with complete medium, cells were plated onto six-well plates pre-coated with a 0.1% gelatin (Sigma) and cultured in adipocyte differentiation medium. To characterize adipocytes, classical Oil Red O (Sigma) staining was performed as described previously with minor modifications [56]. Briefly, cells seeded on six-well plate were fixed in 4% paraformaldehyde solution for 15 minutes at room temperature, and then rinsed twice with distilled water followed by incubation with 60% isopropanol for three minutes. Cells were then stained with 0.5% Oil Red O solution for 30 minutes, washed twice with distilled water and stored in distilled water for imaging. Images were acquired by Zeiss AX10 microscope equipped with a SPOT PURSUIT camera, and analyzed using Image J 1.46p software.

### Differentiation of iPSCs into Adipocytes via MSCs

Mesenchymal stem cells were derived directly from iPSCs as described with minor modifications [57]. The iPS colonies were pretreated with 10 μM ROCK inhibitor Y- 27632 in maintenance medium for 1 hour before dissociation. Colonies were then dissociated into single cells after incubating with collagenase II for 10 minutes in a 37°C incubator. The cells were spun down and re-suspended in the maintenance medium supplemented with 10 μM Y-27632, and seeded onto the 0.1% gelatin-coated 6-well plate at a density of 15,000/cm^2^. After 24 hours, the maintenance medium was supplemented with an equal volume of the derivation medium which contained alpha-MEM (Cellgro), 10% FBS (Life Technologies), 100 nM dexamethasone (Sigma) and 50 μM magnesium L-ascorbic acid phosphate (Sigma). After incubation for two more days, the medium was replaced with the derivation medium and changed every 3–4 days thereafter. At 10 days, the cells were harvested and labeled as passage zero (P0). The cells were expanded on new 0.1% gelatin-coated dish using an expansion medium containing alpha-MEM (Cellgro), 10% Hi-FBS (Life Technologies), 2 mM L-glutamine and 1X non-essential amino acid (both from Life technologies), and were passed at a 1:3 split ratio. For adipogenic differentiation, cells upon subconfluency were induced in adipogenic differentiation medium containing high glucose DMEM supplemented with 10% FBS (both from Life Technologies), 1 μM dexamethasone, 100 μM indomethacin, 500 μM 3-isobutyl-1-methylxanthine (IBMX), and 10 μg/ml insulin (all from Sigma). Lipid production in the 21 day adipogenic cultures was examined with Oil Red O (ORO) staining.

### Spectral karyotype Analysis (SKY)

SKY analysis was performed as described previously [58]. Briefly, twenty cells arrested in metaphase were counted, and five or more metaphases were karyotyped according to the International System for Human Cytogenetics (ISCN; Mitel- man, 1995). For the iPSCs from HGPS-2, all 20 metaphases showed the same abnormal insertions of chromosome 22 on chromosome 2.

### Bodipy 493/503 Staining and Flow Cytometry Analysis

Cells grown on 6-well plate were rinsed with PBS, and then stained with 1 μg/ml bodipy 493/503 in PBS at 37°C for five minutes. Lipid droplets in the cytoplasm were visible as green circular dots under fluorescent microscopy. Stained cells were then harvested by trypsin digestion and washed twice with PBS. Single cell suspensions in 1 ml PBS were prepared by filtration through a 40 μm cell strainer (BD) for FACS analysis (FACSCanto II; BD). Positively stained cells were defined by unstained adipocytes (negative) and the data was further analyzed using FlowJo software (v8.8.8). All conditions were kept same during five weeks of adipocyte differentiation for the dynamical FACS data analysis.

### RNA Extraction, cDNA Synthesis, quantitative RT-PCR and RNA Array

Total RNA from different cell lines was extracted with Trizol (Life Sciences) and purified using the RNeasy Mini Kit (Qiagen) according to the manufacturer's instructions. The RNA yield was determined using the NanoDrop 2000 spectro-photometer. Total RNA (1μg) was converted to cDNA using iScript Select cDNA Synthesis Kit (Bio-Rad). Quantitative RT-PCR was performed in triplicate using SYBR Green Supermix (Bio-Rad) on CFX96 real-time system (C1000 Thermal Cycler; Bio-Rad). All primers used in this study are listed in Table S2.

For PCR array experiments, an RT^2^ Profiler human adipogeneisis PCR array was used to simultaneously examine the mRNA levels of 89 genes, including five “housekeeping genes” in 96-well plates according to the protocol of the manufacturer (SuperArray Bioscience). Each reaction included 1 μg of total RNA and the proper negative controls. RNA of HGPS and normal adipocytes at the fourth week was analyzed in duplicates, and data were normalized with the internal housekeeping genes by the ΔCt method according to the protocol of the manufacturer. Two biological replicates were analyzed.

### Chromatin immunoprecipitation (ChIP) and quantitative ChIP-PCR

Cross-linking reactions of live fibroblasts and iPSCs were carried out in 1% formaldehyde (EMD Chemicals) for 20 minutes at room temperature, then stopped by adding glycine at a final concentration of 0.125 M. Cells were rinsed twice with 1X PBS and harvested using cell scrapers. After centrifuging and flash freezing, cell pellets were stored at −80°C freezer until use. For ChIP experiment, nuclear lysates were prepared by resuspending cell pellets in series of lysis buffers on ice. Chromatins were sheared by sonicating sample lysates 20 seconds for total six times at 30% amplitude with one minute interval on ice (Branson Digital Sonifier 450). Immunoprecipitations (IPs) were performed overnight on the sonicated nuclear extracts with 15 ìg of H3K4me3 antibody (Abcam) pre-coupled to 100 μl Dynal Protein G Magnetic Beads (Life Sciences) at 4°C. After washing precipitates five times in wash buffer, DNA was eluted from beads in elution buffer, then extracted and purified by ethanol precipitation after RNase and proteinase K treatment. Samples used for Western Blotting analysis were obtained from 10% of eluted sample beads by resuspending and boiling in Laemmli Sample Buffer at 95°C for 10 minutes. Quantitative ChIP-PCR was performed in triplicate using SYBR green real-time PCR system (Rio-Rad CFX96) with the following cycling parameters: 95°C for 10 minutes, 40 cycles of 95°C for 15 seconds, 57°C for 15 seconds, and 72°C for 30 seconds. The ChIP data were normalized to 4% input DNA amplifications. The sequences of the *LMNA* promoter primers are listed in Table S2.

### Immunocytochemistry

Immunostaining was carried out using the following antibodies: lamin A/C (MAB3211; Milipore), progerin [23], lamin B (sc-6217; Santa Cruz), Tra-1-81 (560174; BD Pharmingen), Tra-1-60 (560121; BD Pharmingen), SSEA-4 (560219; BD Pharmingen), Nanog (ab21624; Abcam), and Oct4 (ab19857; Abcam). Hoechst 33342 (Life Sciences) or DAPI (Vector Laboratories) was used to counterstain cell nuclei. Images were acquired with either Zeiss AX10 microscope equipped with a SPOT PURSUIT camera or Zeiss LSM 710 confocal microscope.

### Western Blotting Analysis

Whole cell lysates for immunoblotting were prepared by dissolved cells in Laemmli Sample Buffer containing 5% 2-mercaptoethanol (Bio-Rad). Antibodies used in this study included: lamin A/C (sc-6215; Santa Cruz),lamin B (sc-6217; Santa Cruz),GAPDH (ab8245; Abcam), actin (4968; Cell Signaling), PPARγ2 (ab45036; Abcam), C/EBPα (2295; Cell Signaling), GFP (ab290; Abcam), and H3K4me3 (ab1012; Abcam).

### Senescence associated β-Galactosidase activity assay

SA–β-gal activity assay was performed according to the manufacturer's protocol (#9860; Cell Signaling). Briefly, differentiated adipocytes grown on six-well plate were fixed in 1X fixative solution containing 2% formaldehyde and 2% glutaraldehyde for 10 minutes, and then stained overnight at 37°C with the β-galactosidase staining solution at pH 6.0 for 18 hours. Images were acquired by Zeiss AX10 microscope with a SPOT PURSUIT camera.

### Statistical Analysis

Results are presented as mean ± SD. Data were analyzed using 2-tailed Student's t test, and a *p* value less than 0.05 was considered significant.

## SUPPLEMENTAL DATA


