# Supplementary material for: An inhibitory role of progerin in the gene induction network of adipocyte differentiation from iPS cells
Source: Aging (Albany NY). 2013 Apr 14;5(4):288–303. doi: 10.18632/aging.100550 (PMC3651521; doi:10.18632/aging.100550)
Supplement: Supplementary file 1 [file aging-05-288-s001.pdf]

SUPPLEMENTAL DATA

Table S1. iPS cell line information.

Cell line (name used in paper)	Donor Age	Gender
AG08470 (Normal or 8470)	10 yrs	Female
HGADFN164 (HGPS-1 or 164)	4 yrs 8 mos	Female
HGADFN155 (HGPS-2)	1 yr 2 mos	Female
HGFDFN168 (168)	37 yrs	Male, father of 167
HGADFN167 (167)	8 yrs 5 mos	Male

Table S2. Primer sequences.

Gene	Sequence	
LMNA	Sense	5’-GCAACAAGTCCAATGAGGACCA-3’
	Antisense	5’-CATGATGCTGCAGTTCTGGGGGCTCTGGAT-3’
Progerin	Sense	5’-GCAACAAGTCCAATGAGGACCA-3’
	Antisense	5’-CATGATGCTGCAGTTCTGGGGGCTCTGGAC-3’
LMNB 1	Sense	5’-CATGAAACGCGCTTGGTAGA-3’
	Antisense	5’-TTGCGCCAGCTTGTACTCATAC-3’
PPARγ2	Sense	5’-AGGCGAGGGCGATCTTGACAG-3’
	Antisense	5’-GATGCGGATGGCCACCTCTTT-3’
C/EBPα	Sense	5’-GCAAACTCACCGCTCCAATG-3’
	Antisense	5’-TTAGGTTCCAAGCCCCAAGTC-3’
C/EBPβ	Sense	5′-GCGCGAGCGCAACAACA-3′
	Antisense	5′-TGCTTGAACAAGTTCCGCAG-3′
C/EBPδ	Sense	5′-GGTGCCCGCTGCAGTTT-3′
	Antisense	5′-CTCGCAGTTTAGTGGTGGTAAGTC-3′
LMNA promoter	Sense	5’-CACTCCGACTCCGAGC-3’
	Antisense	5’-GTAGACCGCCAAGCG-3’
Tert	Sense	5’-ACTTTGTCAAGGTGGATGTGACGG-3’
	Antisense	5’-AAGAAATCATCCACCAAACGCAGG-3’
Actin	Sense	5’-CTGGAACGGTGAAGGTGACA-3’
	Antisense	5’-AAGGGACTTCCTGTAACAATGCA-3’

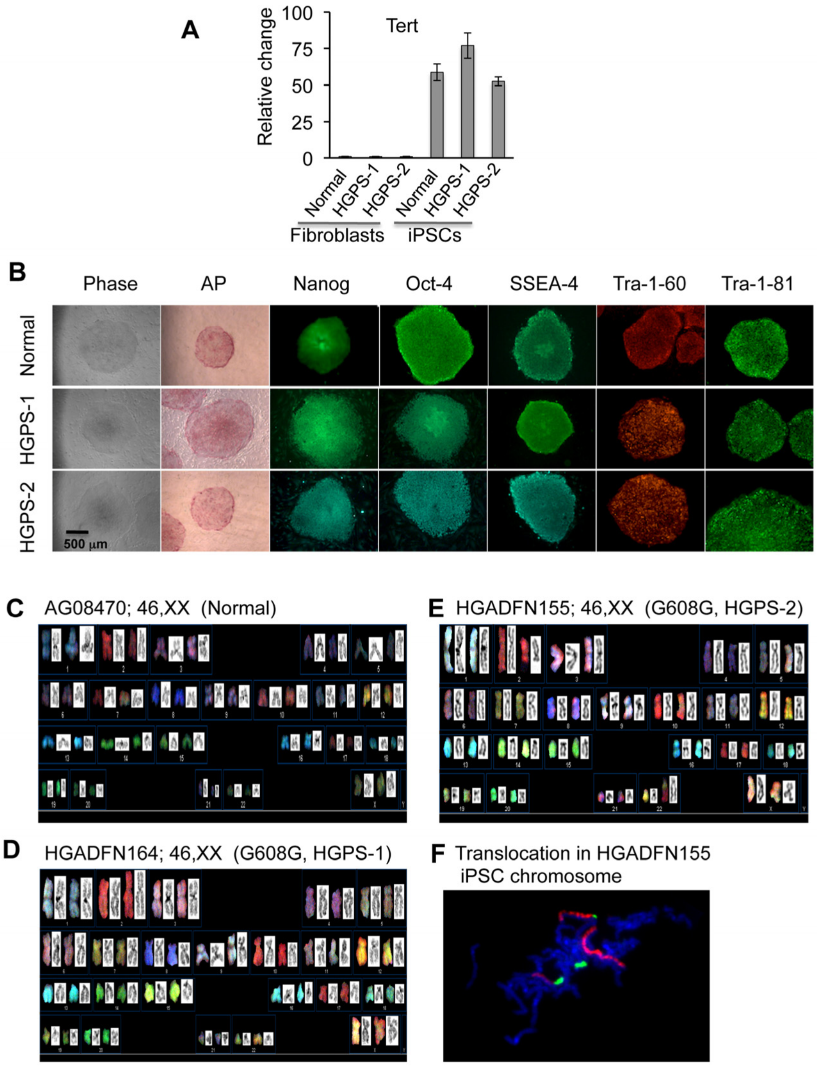

Figure S1. Characterization of iPSCs from normal and HGPS skin fibroblasts. (**A**) Quantitative RT-PCR analysis of Tert mRNA levels in fibroblasts and iPSCs. (**B**) Images of phase contrast, alkaline phosphatase (AP) staining, and immunofluorescence staining with the pluripotency markers: Nanog, Oct-4, SSEA-4, Tra-1-60, and Tra-1-80. Scale bar: 500 µm. (**C-E**) SKY analysis of normal, HGPS-1, and HGPS-2 iPSCs. 20 metaphase cells were examined for each sample, and one representative image is shown. (**F**) Fluorescence *in situ* hybridization image shows insertion of a short segment of chromosome 22 (green) into chromosome 2 (red) in HGPS-2 iPSCs. Cell lines: Normal (8470), HGPS1 (164), and HGPS-2 (155).

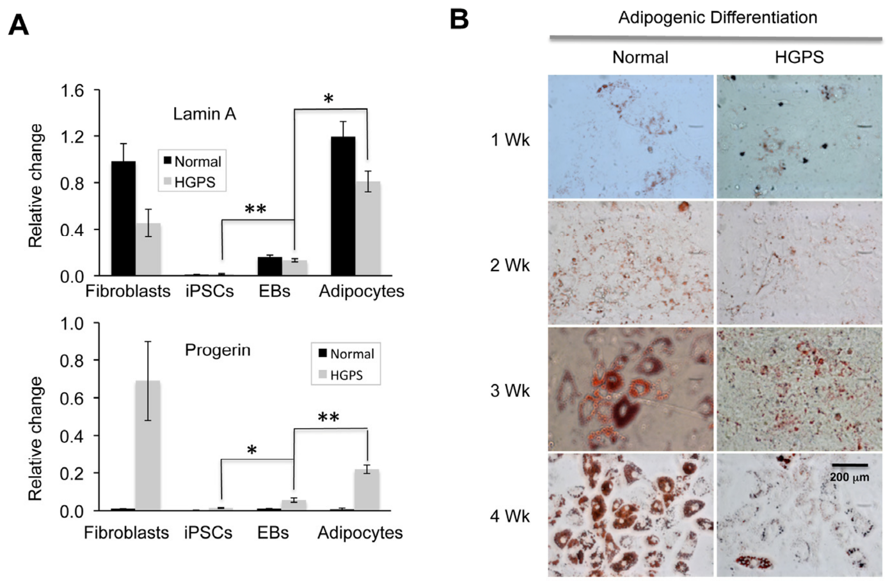

Figure S2. Adipocytes differentiation via EB formaion. (**A**) Quantitative RT–PCR analysis showing the mRNA level of lamin A (upper) and progerin (lower) in normal and HGPS fibroblasts, iPSCs, Ebs, and Adipocytes (n = 3). (* $p < 0.05$; ** $p < 0.01$). (**B**) Oil Red O staining images of normal and HGPS adipocytes during four week differentiation. Scale bar: 200 µm. Cell lines: Normal (8470) and HGPS (164).

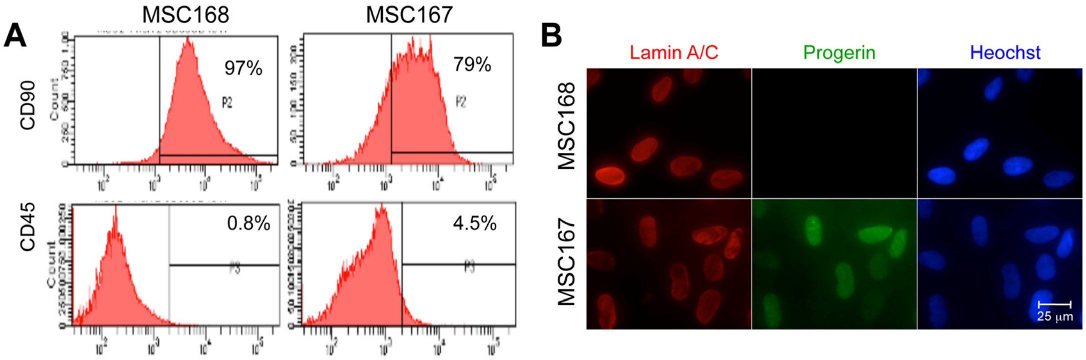

Figure S3. MSC differentiation from iPSCs. (**A**) Sort normal MSC168 and HGPS MSC167 with MSC positive and negative marks CD90 and CD45, respectively. (**B**) Immunostaining images of MSCs with anti-lamin A/C and anti-progerin antibodies. Scale bar: 25 µm.
